# Loss of JAK inhibitor efficacy and subsequent rescue in a pediatric case of alopecia universalis

**DOI:** 10.1016/j.jdcr.2026.02.032

**Published:** 2026-02-21

**Authors:** Ayushya Ajmani, Timothy Klufas, Ishani Rao Dhamsania, Rebecca Yamamoto, Albert E. Zhou

**Affiliations:** a Geisel School of Medicine at Dartmouth, Hanover, New Hampshire; b New York Medical College, School of Medicine, Valhalla, New York; c Department of Dermatology, University of Connecticut, Farmington, Connecticut; d Quinnipiac University School of Medicine, Hamden, Connecticut; e Department of Internal Medicine, John A. Burns School of Medicine, University of Hawai'i, Honolulu, Hawaii

**Keywords:** Alopecia Areata, Alopecia Universalis, Baricitinib, Hair loss, JAK-inhibitor, Loss of efficacy, quality of life, Ritlecitinib, tachyphylaxis

## Introduction

Alopecia Areata (AA) is a chronic autoimmune disease characterized by non-scarring hair loss. The condition has a highly variable course ranging from patchy alopecia to alopecia universalis (AU). Pediatric-onset AA accounts for approximately 18% of all cases, but AU remains rather rare in children and is challenging to treat.^1^ The burden of AU can be profound, particularly in children, affecting quality of life, social development, and mental health.^2^ Janus kinase (JAK) inhibitors have recently emerged as a therapy for moderate to severe AA, with ritlecitinib, a selective JAK3 and tyrosine kinase expressed in hepatocellular carcinoma kinase inhibitor, being approved for adolescents in 2023 by the U.S. Food and Drug Administration (FDA). Although trials have demonstrated efficacy in reducing disease severity and promoting hair growth, limited data are available on the long-term outcomes and relapse among pediatric populations. Moreover, the literature on therapeutic alternatives following JAK-inhibitor failure is limited. Given these knowledge gaps, we present a case of a 10-year-old male with rapidly progressive AU that had an initial profound response to ritlecitinib, but experienced tolerance and a quick relapse within 1 year. The patient demonstrated renewed improvement with baricitinib, a JAK1/2 inhibitor.

## Case presentation

A 10-year-old boy with no significant past medical history presented to his pediatrician with rapid and progressive scalp hair loss. Initial treatment with topical triamcinolone and clobetasol ointment yielded no improvement. Over subsequent weeks, confluent alopecic patches involved the entire scalp, eyebrows, eyelashes, and body, leading to a diagnosis of AU. No precipitating factors, associated systemic symptoms, or environmental exposures were identified at the time. Family history was notable for maternal AA in remission.

**Fig 1:**
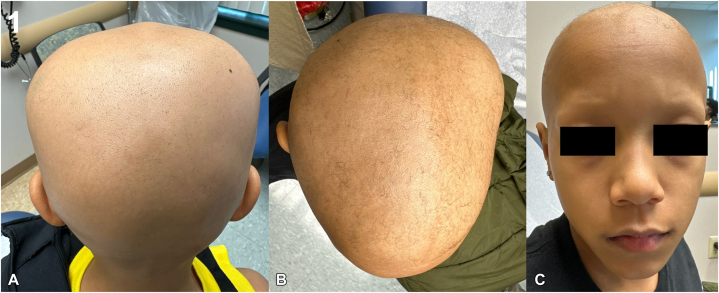
Initial presentation with alopecia universalis. **A,** Posterior scalp showing complete alopecia. **B,** Lateral view with sparse vellus hairs only. **C,** Frontal view demonstrating bilateral madarosis and total scalp hair loss.

**Fig 2:**
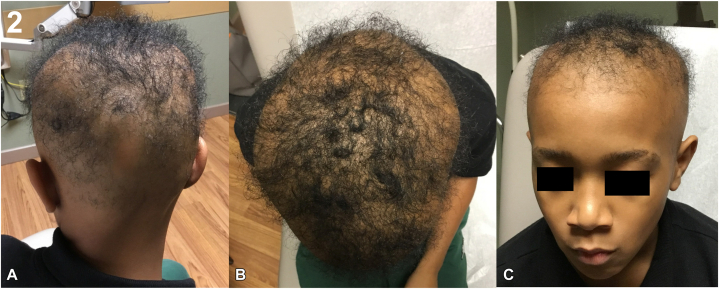
Response after 6 months of ritlecitinib 50 mg daily. **A,** Posterior scalp with significant terminal hair regrowth. **B,** Vertex showing dense central regrowth with residual patches. **C,** Frontal view with partial eyebrow regrowth.

Dermatologic evaluation revealed that the patient had total scalp alopecia, bilateral madarosis, sparse vellus hairs, and diffuse absence of body hair (Fig 1). Laboratory workup, including complete blood count, thyroid-stimulating hormone, inflammatory markers, autoimmune serologies, and viral screenings, was unremarkable, except for a low serum Vitamin D level (26 ng/mL). The patient was initiated on oral ritlecitinib 50 mg daily, vitamin D supplementation, and topical and oral minoxidil, which was titrated up from 1.25 mg to 2.5 mg daily. Treatment was well-tolerated, with gradual regrowth of scalp vellus hairs and partial regrowth of eyebrows and eyelashes over a 6-month period (Fig 2). To address residual patchy alopecia, ruxolitinib 1.5% cream was subsequently prescribed for twice-daily application.

**Fig 3:**
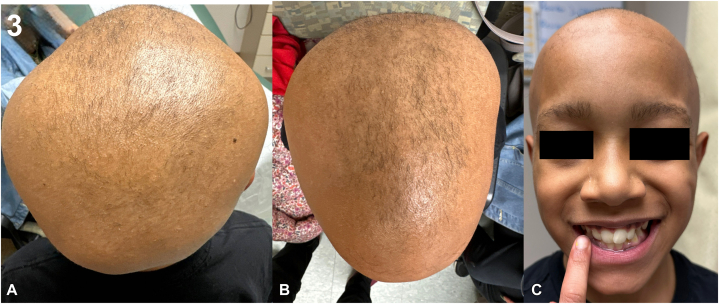
Relapse at 12 months on ritlecitinib. **A,** Posterior scalp showing diffuse-pattern hair loss. **B,** Lateral view with diffuse shedding. **C,** Frontal view with preserved eyebrows but scalp hair loss despite continued therapy.

However, upon nearing the 1-year anniversary of starting ritlecitinib, the patient experienced acute diffuse hair shedding with patchy loss, though sparing the eyebrows. No notable triggers were identified. Examination showed persistent madarosis and failure of regrowth despite continuous ritlecitinib therapy (Fig 3). Monitoring labs remained within normal limits. Given suspected JAK inhibitor failure, the patient was switched to oral baricitinib 4 mg daily, while continuing ruxolitinib 1.5% cream and oral minoxidil.

**Fig 4:**
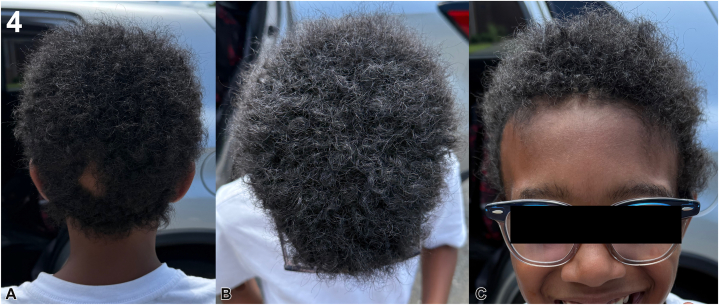
Improvement after switch to baricitinib 4 mg daily after 6 months. **A,** Posterior view with renewed dense hair regrowth. **B,** Vertex demonstrating robust regrowth. **C,** Frontal view showing continued improvement with minimal residual patches.

Following this adjustment, the patient demonstrated renewed growth within 3 months, particularly at the vertex of the scalp. At the most recent 6-month follow-up, the patient remains healthy with significant ongoing improvements in their hair loss with a few patchy areas remaining (Fig 4).

## Discussion

Alopecia areata is mediated by a collapse of immune privilege at the hair follicle, driven by cytotoxic CD8+ natural killer group 2D receptor+ T cells and a cascade of type 1 cytokines, including interleukin-2, interferon gamma, and tumor necrosis factor alpha, factors that orchestrate follicular destruction and disrupt normal hair growth.^3^ These cytokines signal through the Janus kinase – signal transducer and activator of transcription pathway, rendering JAK inhibitors a mechanistically viable target for therapies.

Ritlecitinib, a selective JAK3 and tyrosine kinase expressed in hepatocellular carcinoma family kinase inhibitor, primarily interferes with the gamma-chain cytokine signaling, whereas baricitinib inhibits JAK1 and JAK2, thereby modulating interferon-driven inflammation.^3^^,^^4^ The differential kinase selectivity of these agents has not been shown to translate into differences in efficacy and safety. Although ritlecitinib is not presently FDA-approved for use in children under 12 years of age, limited data suggests it is well tolerated in patients aged 6 to 12 years.^5^ In contrast, baricitinib has received regulatory approval outside of the United States, including in the European Union, for pediatric use in patients as young as 2 years of age with atopic dermatitis.^6^

Our patient exhibited an initial therapeutic response to ritlecitinib, followed by an abrupt hair shedding in a diffuse pattern despite adherence. The clinical course raised concern for JAK inhibitor failure, natural disease cycling, evolution of disease to an alternative subtype, or the emergence of tachyphylaxis. Although traditionally described in the context of corticosteroid therapies, tachyphylaxis with JAK inhibitors may reflect receptor desensitization, downstream signaling changes, or even the upregulation of compensatory inflammatory pathways.^7^ Nonetheless, these phenomena are sparsely documented within existing literature.^8^, ^9^, ^10^

Switching from ritlecitinib to baricitinib was associated with disease stabilization and renewed hair growth for our patient, suggesting that intra-class switching may confer benefits to patients with non-response.^8^, ^9^, ^10^ Recent evidence suggests patients may demonstrate early, gradual, or late-responder phenotypes to JAK inhibitors.^4^

As ritlecitinib remains the only FDA-approved JAK inhibitor for adolescent AA, loss of efficacy is concerning. Recent reports have documented successful JAK inhibitor switching in predominantly adult cohorts (Table I). While recent literature has begun to elucidate pediatric considerations, these data have been limited.^2^ This case illustrates successful disease stabilization and hair regrowth following switch from ritlecitinib to baricitinib in a pediatric patient with AU who experienced treatment failure after 1 year. Clinical improvement occurred within 3 months, with sustained response at follow-up, suggests that failure of 1 JAK inhibitor does not preclude response to another in pediatric populations.

**Table I:** Literature review of JAK inhibitor efficacy after initial JAK inhibitor failure in patients with alopecia areata

Article #	Citation	Patient population	Study description
1	Ehsani A, Razavi Z, Rahimnia A, et al. Switching JAK inhibitors: evaluating baricitinib's effectiveness in alopecia areata after tofacitinib failure. Archives of Dermatological Research. 2025;317:491. https://doi.org/10.1007/s00403-025-04035-y	26 patients, 65% female, Mean age 31 y	Retrospective study of 26 AA patients switched from tofacitinib to baricitinib. SALT reduced from 90 ± 19 to 68 ± 31 with tofacitinib (16 mo), then 68 ± 31 to 50 ± 31 with baricitinib (7 mo). 66.66% of tofacitinib non-responders improved with baricitinib. Adverse effects: 30.76% on tofacitinib vs 3.84% on baricitinib.
2	van Helmond SC, Willaert M, Nguyen VH, et al. Real-world effectiveness and safety of Janus kinase inhibitors in alopecia areata: a retrospective cohort study of 72 patients. Acta Derm Venereol. 2025;105:adv42990. https://doi.org/10.2340/actadv.v105.42990	72 patients, 72.2% female, mean age 34 y	Retrospective cohort of 72 AA patients treated with various JAKi. Mean disease duration 8 y, median baseline SALT 100. Over median 16-month follow-up, 61% achieved substantial regrowth at median 7 months. Cumulative regrowth rates: 11.1% (3 mo), 40.2% (6 mo), 55.6% (9 mo), 59.7% (12 mo). Among non-responders who switched JAKi, 75% achieved substantial regrowth.
3	Jiminez V, Riess A, Mayo T, Elewski B. Alopecia universalis: never give up? J Drugs Dermatol. 2025;24(3):316-318. https://doi.org/10.36849/JDD.8587	1 patient, female, age 28 y	28-year-old female with AU (SALT 100%) failed 1 y baricitinib, switched to ritlecitinib 50 mg daily. After 6 mo: vellus hair regrowth. After 1 y ritlecitinib (2 total years JAKi): robust terminal hair growth with SALT 17.2. Patient had Turner's syndrome, hypothyroidism, atopic dermatitis. Demonstrates importance of prolonged JAKi therapy and intra-class switching.
4	Kalil L, Craiglow BG, King B. Successful treatment of alopecia areata with 1 Jak inhibitor after failure of other JAK inhibitors. JAMA Dermatology. Published online October 8, 2025. https://doi.org/10.1001/jamadermatol.2025.3537	13 patients, predominantly male, white (some African Americans, and Hispanic/Latino)	Case series of 13 severe AA patients who failed 1 or more JAKi (minimum 6 mo each) before achieving SALT ≤20 with different JAKi. Patients switched between tofacitinib, baricitinib, ritlecitinib, upadacitinib, ruxolitinib, and deuruxolitinib. Demonstrates failure of 1 JAKi does not predict failure of another.
5	Martin A, Chen LC, Kreytak C, et al. Switching between Janus kinase inhibitors for treatment of alopecia areata: A multicenter retrospective review. Journal of the American Academy of Dermatology. 2026;0(0). https://doi.org/10.1016/j.jaad.2026.01.055	108 patients, 66.7% female, Mean age 38.5 y	Multicenter retrospective review of 108 patients who switched oral JAKis after a minimum treatment period of 6 months. 48.8% achieved a SALT score of ≤20 on their second JAK inhibitor; 24 patients had to move to a third JAKi. Strongest predictor of success on a second JAKi was whether the patient had responded to the first 1.

*JAK*, Janus kinase; *JAKi*, Janus kinase inhibitor; *SALT*, Severity of Alopecia Tool

Another challenge in management is the absence of standardized, time-based outcome metrics. The Severity of Alopecia Tool quantifies disease extent through temporal parameters, including time-to-onset of regrowth, duration of response, or time-to-relapse.^1^^,^^4^ Additionally, the lack of validated biomarkers or operational definitions of therapeutic success versus failure complicates interpretation, making it difficult to distinguish true treatment response from natural disease cycling.^4^

As JAK inhibitors become increasingly prevalent, further research is needed to elucidate mechanisms of therapeutic resistance. Early, proactive, and transparent discussions acknowledging both the potential benefits and limitations of JAK inhibition can help prepare patients and families for variable clinical courses, including time-to-response, relapse, or future therapeutic adjustments.

## Conflicts of interest

None disclosed.
